# R-loop Mediated DNA Damage and Impaired DNA Repair in Spinal Muscular Atrophy

**DOI:** 10.3389/fncel.2022.826608

**Published:** 2022-06-16

**Authors:** Juliana Cuartas, Laxman Gangwani

**Affiliations:** ^1^Center of Emphasis in Neurosciences, Department of Molecular and Translational Medicine, Paul L. Foster School of Medicine, Texas Tech University Health Sciences Center El Paso, El Paso, TX, United States; ^2^Francis Graduate School of Biomedical Sciences, Texas Tech University Health Sciences Center El Paso, El Paso, TX, United States

**Keywords:** R-loops, NHEJ, genomic instability, SMA, neurodegeneration, ALS, ZPR1, senataxin

## Abstract

Defects in DNA repair pathways are a major cause of DNA damage accumulation leading to genomic instability and neurodegeneration. Efficient DNA damage repair is critical to maintain genomicstability and support cell function and viability. DNA damage results in the activation of cell death pathways, causing neuronal death in an expanding spectrum of neurological disorders, such as amyotrophic lateral sclerosis (ALS), Parkinson’s disease (PD), Alzheimer’s disease (AD), and spinal muscular atrophy (SMA). SMA is a neurodegenerative disorder caused by mutations in the *Survival Motor Neuron 1* (*SMN1*) gene. SMA is characterized by the degeneration of spinal cord motor neurons due to low levels of the SMN protein. The molecular mechanism of selective motor neuron degeneration in SMA was unclear for about 20 years. However, several studies have identified biochemical and molecular mechanisms that may contribute to the predominant degeneration of motor neurons in SMA, including the RhoA/ROCK, the c-Jun NH_2_-terminal kinase (JNK), and p53-mediated pathways, which are involved in mediating DNA damage-dependent cell death. Recent studies provided insight into selective degeneration of motor neurons, which might be caused by accumulation of R-loop-mediated DNA damage and impaired non-homologous end joining (NHEJ) DNA repair pathway leading to genomic instability. Here, we review the latest findings involving R-loop-mediated DNA damage and defects in neuron-specific DNA repair mechanisms in SMA and discuss these findings in the context of other neurodegenerative disorders linked to DNA damage.

## Introduction

The maintenance of genomic integrity is critical for the survival and propagation of life on earth. All cells must protect their genomes from the normal wear and tear caused by DNA replication, gene transcription, ongoing metabolic reactions that produce DNA damaging compounds, as well as physical and chemical stresses from the external environment. As a result, cells have evolved a dizzying array of proteins that function in all aspects of nucleic acid metabolism and evolved DNA repair pathways to manage different types of genomic insults. The accumulation of DNA damage that leads to genomic instability is toxic for cells, triggering downstream reactions which can result in cell death if the damage is not repaired in a timely manner. The discovery of DNA damage and endogenous DNA repair pathways in living organisms in the last century introduced a new milestone in the field of cellular and molecular biology. These advances allowed examining and understanding the molecular basis of human diseases caused by DNA damage, including neurological and neurodegenerative illnesses (Branzei and Foiani, 2008; Saini, 2015; Chatterjee and Walker, 2017).

Neurons are complex and can be physically massive cells: the longest neurons in the human body span the length of the leg. Neurons are terminally differentiated cells that do not replicate their genomes and must survive the life of the organism. Neurons with long and branching axons such as motor neurons require constant maintenance and specialized structural upkeep and require the expression of large amounts of highly specialized protein products. The high levels of transcription and translation of proteins require increased levels of energy expenditure and metabolism, which produces DNA damaging compounds such as reactive oxygen species (ROS). An increase in transcription levels directly correlates with the increase in DNA damage accumulation that could result in unmanageable genomic instability (D’Alessandro and d’Adda Di Fagagna, 2017).

In this review, we will discuss two major causes of genomic instability leading to neurodegeneration: R-loop accumulation, and defects in DNA damage response (DDR) pathways. We will briefly introduce the spectrum of neurodegenerative and neurological illnesses that are associated with defects in R-loop homeostasis and DNA repair pathways. We then focus on the advances made in the field of spinal muscular atrophy, a neurodegenerative disease of early childhood, and discuss in detail how and why altered R-loop resolution and impaired DNA repair pathways contribute to genomic instability and predominant degeneration of motor neurons. Further, we will discuss studies that have provided insight into R-loop resolution complexes and the mechanism of R-loop resolution, including studying using two motor neuron disease models SMA and amyotrophic lateral sclerosis 4 (ALS4) with opposite alterations in R-loop metabolism.

## R-Loop Accumulation, Genomic Instability, and Diseases of The Central Nervous System

**Figure 1:**
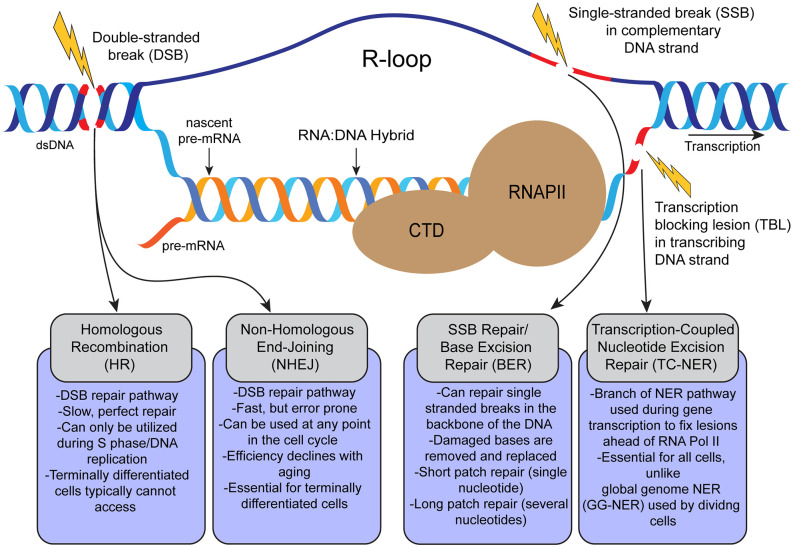
Schematic representation of formation of transcription-coupled R-loops and possible DNA lesions that occur during transcription and related DNA repair pathways in the cell. R-loops are 3-strand nucleic acids structures formed during RNA Pol II (RNAPII)-dependent gene transcription. R-loops are composed of a nascent mRNA transcript hybridized to the transcribing DNA strand, and the displaced complementary DNA strand. Persistent pathogenic R-loops can result in DNA lesions such as single-stranded breaks (SSBs) and double-stranded breaks (DSBs) by various mechanisms. From left to right, DSBs can be repaired *via* homologous recombination (HR; in dividing cells), or non-homologous end-joining (NHEJ; in dividing and terminally-differentiated cells). Single stranded breaks (SSBs) can be repaired by base excision repair (BER). Transcription blocking lesions (broadly) are repaired through transcription-coupled nucleotide excision repair (TC-NER).

High levels of transcription in neurons increase the levels of R-loops, which increase the risk of DNA damage if these are not promptly resolved. R-loops are naturally occurring RNA:DNA hybrids that form during transcription and are composed of three nucleic acid strands, a DNA coding (transcribing) strand hybridized to nascent pre-mRNA, and a displaced non-coding (complementary) DNA strand (Figure 1). R-loops are one potential avenue of DNA damage in an expanding spectrum of disorders (Groh and Gromak, 2014; Garcia-Muse and Aguilera, 2019) including neurological and neurodegenerative disorders such as ataxia telangiectasia (AT; Shiloh and Rotman, 1996), ALS (Salvi and Mekhail, 2015), ataxia oculomotor apraxia 2 (AOA2; Fogel et al., 2014; Becherel et al., 2015), and spinal muscular atrophy (SMA; Kannan et al., 2018; Hensel et al., 2020). Additionally, R-loop mediated genomic instability and downstream DNA damage may be implicated in other disorders, including Fanconi anemia and related illnesses, which display neurological symptoms but are not classified as neurological disorders (Okamoto et al., 2019b). R-loops are also considered a hallmark of cancer, present in various leukemias, and thought to aid in tumor progression in breast cancer (Richard and Manley, 2017).

R-loops are formed during transcription, the non-coding DNA strand is displaced as the coding strand is transcribed and forms a hybrid with the nascent pre-mRNA. The nascent pre-mRNA must be released from the transcribing DNA strand to be processed and spliced into mRNA. However, alterations in the timely processing of R-loops could affect the splicing or alternative splicing of pre-mRNA (Li and Manley, 2005). R-loops are a ubiquitous feature of transcription and form across the genomes of most known living prokaryotic and eukaryotic organisms (Crossley et al., 2019). Much effort has gone into contextualizing R-loops: these inherent byproducts of transcription can play physiological as well as pathological roles (Richard and Manley, 2017; Garcia-Muse and Aguilera, 2019; Hegazy et al., 2020; Mackay et al., 2020).

R-loops may regulate transcription at promoter sequences, block transcription factor binding in a potential negative feedback loop mechanism (Skourti-Stathaki and Proudfoot, 2014; Grunseich et al., 2018; Niehrs and Luke, 2020), and contribute to DNA repair (Ohle et al., 2016; Lu et al., 2018; Marnef and Legube, 2021). Whether there is a structural, or sequence-specific difference between beneficial or regulatory R-loops and pathological R-loops remains to be investigated. Another hypothesis that has been proposed is that cells can tolerate R-loop accumulation up to a certain threshold, after which the R-loop accumulation becomes pathological and leads to genomic instability.

In dividing cells, collisions between replication and transcription machinery can cause single-stranded breaks (SSBs) and double-stranded breaks (DSBs), and trigger the DNA damage response through activation of proteins ataxia telangiectasia mutated (ATM), and ataxia telangiectasia-related (ATR). In cycling cells (including neural progenitor cells in the developing nervous system), these collision damages can be fixed by homologous recombination (HR) and non-homologous end joining (NHEJ; Orii et al., 2006). Another source of DNA damage is R-loop accumulation during transcription. Accumulation of unresolved R-loops or DNA damage ahead of the transcription machinery can lead to stalling of RNA Pol II (RNAPII; Sepe et al., 2013; Lans et al., 2019). The stalling of RNA Pol II in response to DNA lesions may also result in further accumulation of R-loops. Accumulation of R-loops in response to the loss or inhibition of RNA biogenesis factors such as Aquarius (AQR), SETX, and topoisomerase I result in DSBs by the recruitment of transcription-coupled nucleotide excision repair (TC-NER) factors XPF and XPG. In addition, R-loop-dependent DSB formation requires TC-NER factor Cockayne Syndrome group B (CSB) but not global genome repair (GG-NER) factor XPC suggesting that TC-NER factors may play a negative role and promote DSB formation (Sollier et al., 2014; Hamperl et al., 2017; Cristini et al., 2019).

R-loop-mediated DNA damage may contribute to the pathogenesis of different forms of ALS caused by mutations in the *superoxide dismutase 1* (*SOD1*), *open reading frame 72 at chromosome 9* (*C9ORF72*), *fused in sarcoma (FUS)*, and *transactivation response DNA binding protein, 43*kDa (*TDP-43*) genes. However, further studies are required to gain mechanistic insights into the correlation between DNA damage, impaired DNA repair, and ALS pathogenesis (Salvi and Mekhail, 2015; Wang et al., 2015; Perego et al., 2019). Notably, recent studies have shown that deficiency or loss-of-function of TDP-43 results in an increase of R-loops and accumulation of DNA damage, due to defects in DNA-PKcs-mediated NHEJ repair in cells/neurons lacking TDP-43 or ALS10 patient cells that have TDP-43 mutations (Mitra et al., 2019; Giannini et al., 2020; Konopka et al., 2020).

The low levels of R-loops found in ALS4, an autosomal dominant disease caused by mutations in the *senataxin* (*SETX*) gene (Chen et al., 2004), present an interesting contrast with diseases like SMA, which are characterized by high levels of R-loops. SETX is an ATP-dependent RNA/DNA helicase that unwinds RNA:DNA hybrids and contributes to R-loop resolution (Skourti-Stathaki et al., 2011; Bennett and La Spada, 2018; Cohen et al., 2018). Autosomal dominant characteristics of ALS4 and studies with patient-derived fibroblasts that have L389S mutation in SETX show reduced R-loop accumulation, suggesting a SETX-dependent gain-of-function in R-loop resolution (Fogel et al., 2014; Grunseich et al., 2018). How exactly the low levels of R-loops contribute to neurodegeneration in ALS4 is unclear. Based on findings from ALS4 patient-derived mitotic cells, it is proposed that R-loops promote transcription by blocking DNA methylation at promoters (Grunseich et al., 2018). Reduced R-loops cause downregulation of BMP (bone morphogenic protein) and activin membrane-bound inhibitor (BAMBI), which is a negative regulator of transforming growth factor-β (TGF-β) signaling pathway. Previous studies have also suggested the role of SMN-dependent BMP/TGF-b signaling in the formation and growth of neuromuscular junctions in ALS and SMA (Chang et al., 2008; Bayat et al., 2011). These findings suggest that increased TGF-β signaling may contribute to neurodegeneration in ALS4 (Grunseich et al., 2018). Studies with two ALS4 mouse models with mutations in SETX (L389S and R2136H) and spinal cord tissues from patients show nuclear exclusion as well as cytoplasmic mislocalization of TDP-43. TDP-43 mislocalization to the cytoplasm is a hallmark pathology reported in ALS patients, suggesting that impaired nucleocytoplasmic trafficking in ALS4 may be a cause of motor neuron degeneration in ALS4 (Bennett et al., 2018). Another, not mutually exclusive, possibility is that due to the regulatory, feedback, repair, and other potential roles played by both sequence-specific and R-loops writ large, a tissue-specific basal level of R-loops must be maintained for cellular homeostasis. The reduced R-loop levels could contribute to transcription-related cellular stress in post-mitotic neurons, causing a deregulation of the DNA repair axis and leading to selective degeneration of motor neurons in ALS4, but this hypothesis warrants further studies.

The cellular threat associated with R-loop accumulation to long-lived, non-dividing (post-mitotic), and transcriptionally “high maintenance” cells such as neurons, could stem from a progressive accumulation of R-loops leading to SSBs and DSBs. Existing SSBs and DSBs in highly transcribed genes can generate transcriptional stress and increase the accumulation of R-loops caused by RNA Pol II stalling. TC-NER-mediated R-loop processing used to minimize transcriptional stress can create further DNA damage and R-loop accumulation (Lin and Wilson, 2012; Lans et al., 2019; Nakazawa et al., 2020). R-loops seem to beget more R-loops, and DNA damage, creating a cascade effect which results in a greater load of DSBs to repair. Notably, neurons must rely primarily on NHEJ-mediated DSB repair, which, as discussed, may not be effective enough to repair the DNA damage encountered by neurons under cellular and genomic stress. The NHEJ-mediated DNA repair pathway also gradually loses efficiency with aging, highlighting the potential contribution of age-related DNA damage accumulation and inefficient DNA repair to neurodegenerative disorders such as Alzheimer’s disease (AD; Lin et al., 2020).

## Defects in DNA Repair and Neurological Disorders

Defects in genomic integrity and downstream DNA repair are implicated in a spectrum of neurodegenerative disorders, including amyotrophic lateral sclerosis (ALS; Walker and El-Khamisy, 2018; Konopka et al., 2020; Wood et al., 2020) and Alzheimer’s disease (AD; Kanungo, 2013; Hegde et al., 2017; Wang et al., 2017; Rao et al., 2018). Genomic instability caused by defects in DNA repair is implicated in the downstream neurological consequences of disorders such as Fanconi anemia and Nijmegen Breakage Syndrome, which are known to result in congenital microcephaly (Kastan, 2008; van der Lelij et al., 2010; Okamoto et al., 2019a). Dozens of genes, including *Nijmegen Breakage Syndrome 1 (NBS1), ATM, ATR*, *xeroderma pigmentosum* (*XP*), and *CSB* (among others, reviewed in Barzilai et al., 2008; O’Driscoll and Jeggo, 2008), which have functions in DNA damage signaling, activation of DDR pathways, and SSB and DSB repair pathways, have been characterized only as a result of their association to neurological diseases, including early and late onset.

Evidence suggests that DDR pathway factors and other genes that function in the maintenance of genome integrity play key roles in the early development of the CNS. Disruption of core proteins (XRCC4 or Ligase IV) in the NHEJ-mediated DNA repair pathway results in embryonic lethality due to increased neuronal apoptosis, but only in the presence of P53 (reviewed in Barzilai et al., 2008; McKinnon, 2009). This interaction suggests that P53-dependent cell death may contribute to neurodegeneration. Ligase IV/P53 knockout mice, which accumulated DNA damage and developed a neurodegenerative phenotype later in life, suggest that the accumulation of DNA damage led to different consequences depending on the stage of neural differentiation. Neurons that would normally have been pruned due to high levels of DNA damage were able to differentiate and live in the absence of P53-mediated signaling but eventually succumbed to degeneration later in life, as the load of DNA damage increases (Barnes et al., 1998; Frank et al., 2000).

Efficient DDR is crucial for the survival and homeostasis of terminally differentiated neurons as well (Hegde et al., 2017; Walker and El-Khamisy, 2018). As discussed, terminally differentiated neural cells have limited double-stranded break repair options (Rass et al., 2007; Kannan et al., 2018). When DSBs arise in neurons, whether as a result of R-loop-mediated DNA damage, or other causes, these must be repaired efficiently to make genomic DNA ready for the next round of transcription. Terminally differentiated cells, such as neurons, have limited DNA DSB repair options, and must rely on mistake-prone NHEJ-mediated DSB repair, focused primarily on highly transcribed genes. By contrast, proliferating cells, such as neural progenitor cells can ensure error-free DSB repair using the HR pathway in the S-phase of the cell cycle (Mao et al., 2008). The efficiency of NHEJ-mediated DNA repair declines with aging, and defects in various factors in the pathway result in an increased risk of DNA damage accumulation leading to genomic instability and varying severities of neurodegenerative phenotypes (Barzilai, 2007; Rao, 2007; Rass et al., 2007; Kannan et al., 2018; Konopka et al., 2020). One critical factor required for NHEJ-mediated DNA repair is the DNA-dependent protein kinase catalytic subunit (DNA-PKcs). Downregulation of DNA-PKcs levels or catalytic activity may contribute to the pathogenesis of neurodegenerative diseases such as AD (Kanungo, 2016; Lin et al., 2020) and motor neuron disorders ALS and SMA as discussed below.

Neurons are not only more vulnerable to DNA damage accumulation due to oxidative and transcriptional stress but are also challenged by inefficient repairing of DNA lesions that arise and accumulate during their lifespan. In long-lived neurons, this causes genomic instability leading to neurodegeneration and eventually the activation of cell death pathways. This connection may shed light on the link between genomic instability and age-related neurodegeneration. Higher levels of DNA damage and defects in NHEJ-mediated DNA repair have been reported in AD, PD, and ALS as compared to age-matched controls, indicating a strong association between age-related neurodegenerative diseases and accumulation of DNA damage (reviewed in Madabhushi et al., 2014; Walker and El-Khamisy, 2018). Accumulation of DNA damage in the developing nervous system leads, on the other hand, to many of the early onset congenital microcephalies. The common factor seems to be the accumulation of DNA damage leading to genomic instability and resulting in neurodegeneration and cell death, or early cell death leading to microcephaly. SMA, as a genomic instability-mediated neurodegenerative illness of early childhood provides particularly useful insights.

## Spinal Muscular Atrophy

SMA is the most common genetic cause of death in early childhood and the second most common autosomal recessive disorder in humans (Wirth, 2021). SMA is an autosomal recessive neurodegenerative disease caused by mutations of the *survival motor neuron 1* (*SMN1*) gene located at 5q13. The *SMN2* copy in humans produces a truncated version of the SMN protein (SMNΔ7) and a small amount (~10%) of full-length SMN protein which is insufficient and results in degeneration of motor neurons leading to the development of muscle atrophy (Lorson et al., 1999; Ahmad et al., 2016). The number of *SMN2* copies present is inversely correlated with disease severity and positively correlated with age of onset, and patients with six or more copies of *SMN2* do not experience neurodegenerative symptoms until their 30s (Markowitz et al., 2012). Chronic low levels of SMN, a multifunctional protein with a variety of roles in mammalian cells, result in the degeneration of spinal motor neurons, causing muscle atrophy that is followed by symmetric limb paralysis and respiratory distress (Ahmad et al., 2016; Singh et al., 2017).

Clinical investigations of patients have suggested that SMA is a systemic disorder, affecting many other cell and tissue types in addition to the spinal cord motor neurons. Insurance claims filed by SMA patients and families that have received treatment report complaints involving various body systems, implying that a true SMA disease rescue would target cells outside the central nervous system and reduce global defects caused by SMN insufficiency (Shababi et al., 2014; Lipnick et al., 2019). Studies with mouse models also support the idea that SMA is not simply a motor neuron disease (Kim et al., 2020). That SMA is a systemic disorder is evidenced by the fact that SMN is ubiquitously expressed in all cell types and chronic SMN-deficiency may contribute to cell type-specific defects in SMA. Why SMA neurons (especially motor neurons) seem to be “selectively or predominantly” vulnerable to the effects of SMN deficiency has been a topic of intense research and debate. The initial hypothesis on the selective degeneration of motor neurons in SMA was based on the role of the SMN complex in the assembly of spliceosomes and pre-mRNA splicing, and it was thought that defects in the splicing of neuron-specific genes may contribute to selective degeneration (Gubitz et al., 2004; Monani, 2005; Burghes and Beattie, 2009). However, neuron-specific genes that were not also altered in other tissues were not identified, leaving the question open in the SMA field (Zhang et al., 2008). Interestingly, downregulation of genes *AGRIN* and *ETV1*, associated with synaptogenesis, a neuron-specific process, may contribute to the degeneration of neurons in SMA (Zhang et al., 2013). With no neuron-specific candidate genes identified, a new hypothesis was proposed: defects in cellular processes on which neurons depend may be the underlying cause of selective or predominant motor neuron degeneration in SMA (Kannan et al., 2018).

## DNA Damage in SMA

**Figure 2:**
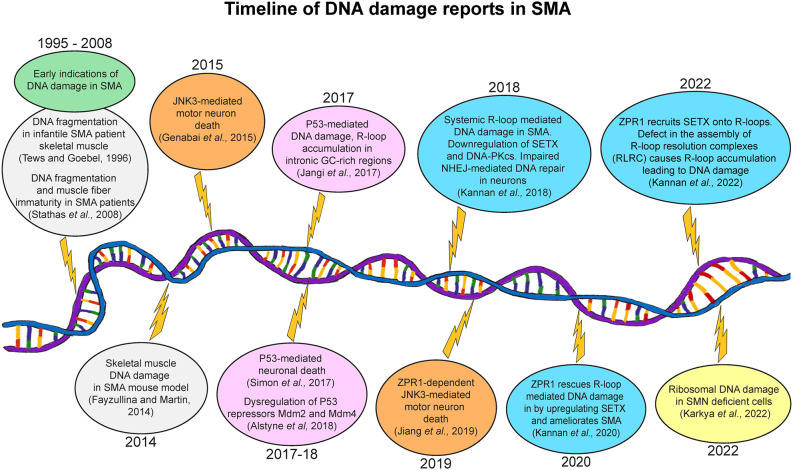
Graphical representation of timeline of DNA damage reports in SMA. Early reports indicating the possibility of DNA damage in SMA muscle tissue (1996–2008) followed by substantial reports with studies uncovering the molecular basis of R-loop-dependent DNA damage in the pathogenesis and the rescue of SMA disease (2014–2022).

DNA damage was initially reported in SMA patient muscle tissues and fibroblasts using simple experiments such as TUNEL assay and electrophoretic analysis of genomic DNA for the presence of DNA lesions/fragmentation (Tews and Goebel, 1996; Stathas et al., 2008; Fayzullina and Martin, 2014). Studies with chemical or radiation-induced DNA damage proposed that faulty DNA repair was unlikely to contribute to SMA pathogenesis (Fayzullina and Martin, 2016). The early notion that SMA is a motor neuron disease, and the lack of convincing biochemical data on the involvement of DNA damage and repair pathways contributed to the paucity of research on the role of genomic instability in SMA pathogenesis. However, convincing experimental evidence has emerged in the past 5 years demonstrating the critical role of DNA damage in SMA pathogenesis. These findings include: the presence of P53-mediated DNA damage *via* dysregulation of P53 repressors Mdm2 and Mdm4 (Jangi et al., 2017; Simon et al., 2017; Van Alstyne et al., 2018), R-loop accumulation and R-loop-mediated DNA damage (Zhao et al., 2016; Jangi et al., 2017; Kannan et al., 2018), rescue of R-loop-mediated DNA damage (Kannan et al., 2020) and defects in the assembly of R-loop resolution complexes (RLRC) as a cause of nuclear/nucleolar R-loop accumulation and DNA damage in SMA (Kannan et al., 2022). A recent study shows that the knockdown of DDX21 may contribute to nucleolar R-loop accumulation and ribosomal DNA damage in SMN-deficient cells (Karyka et al., 2022). A timeline for the involvement of DNA damage and impaired DNA repair in SMA pathogenesis is shown in Figure 2.

**Figure 3:**
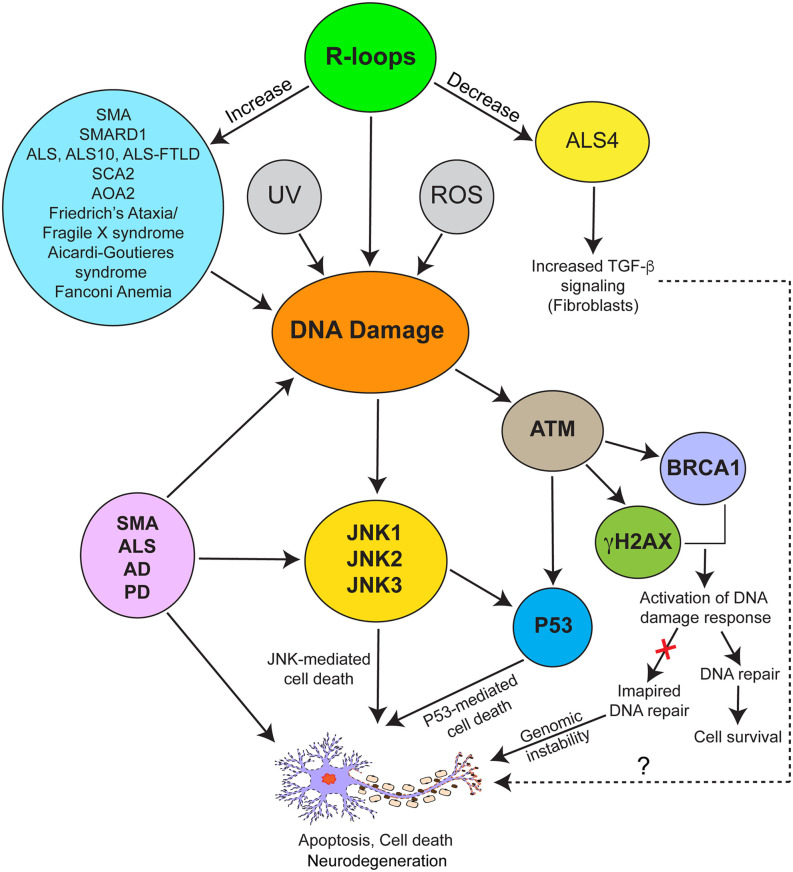
Schematic representation of biochemical signaling mechanisms involved in mediating pathogenic effects of R-loop-mediated DNA damage in neurodegeneration associated with neurological disorders. SMA and various disorders (blue ellipse) are associated with increased R-loops (green ellipse) and DNA damage. R-loop accumulation, UV irradiation, and presence of reactive oxygen species (ROS) are among other causes of DNA damage. ALS4 (yellow ellipse) represents a special example of neuromuscular disorder caused by decrease in levels of R-loops, which is in contrast to SMA caused by increase in R-loop levels compared to controls. DNA damage response is mediated by activation of the JNK, ATM, and P53-mediated signaling pathways. ATM mediates phosphorylation of H2AX (γH2AX) and BRCA1 leading to activation of DNA damage and repair response. Both ATM and JNK phosphorylate P53, which may independently or synergistically contribute to P53-mediated cell death in response to DNA damage. Activation of the JNK pathway contributing to neurodegeneration has been reported in SMA, various forms of ALS, AD, and PD.

The cellular signaling pathways that are activated in response to DNA damage and are reported to be involved in mediating neuronal cell death or apoptosis in neurological disorders, including SMA are shown in Figure 3. The c-Jun NH_2_-terminal kinase (JNK) pathway has been implicated in neurodegeneration associated with diseases such as ALS, AD, PD, and SMA (Schellino et al., 2019). The JNK pathway may mediate the DNA damage response in P53-dependent and independent ways (Fuchs et al., 1998; Picco and Pages, 2013). Two signaling modules, ASK1/MKK4/7/JNK and MEKK1/MKK4/7/JNK leading to JNK activation were identified in SMA patient and mice spinal cords (Genabai et al., 2015). Knockdown of zinc finger protein 1 (ZPR1), which is downregulated in SMA (Helmken et al., 2003; Ahmad et al., 2012), causes JNK-mediated degeneration of cultured primary spinal cord motor neurons derived from mice (Jiang et al., 2019). DNA damage-mediated JNK activation results in the phosphorylation of H2AX and initiation of the DNA damage response. JNK interacts with P53 and directly phosphorylates P53 resulting in P53-mediated apoptosis. Interestingly, DNA damage-mediated activation of ATM results in P53 phosphorylation at Ser-15 (human) and Ser-18 (mouse) and disruption of Mdm2-P53 complexes resulting in P53-mediated cell death (Chao et al., 2000; Picco and Pages, 2013). In SMA, phosphorylation of P53 (Ser-18) and dysregulation of Mdm2 and Mdm4, repressors of P53, results in induction of P53-mediated neuron degeneration (Simon et al., 2017; Van Alstyne et al., 2018). In addition, other signaling pathways, including RhoA/ROCK pathway may also contribute to stress-fiber mediated DNA damage pathogenesis in SMA but requires further investigations (Bowerman et al., 2007; Nolle et al., 2011; Cheng et al., 2021; Magalhaes et al., 2021). The evidence above indicates that DNA damage accumulation in SMA neurons leads to the activation of cell death pathways, but the source of the DNA damage remained unclear.

## R-Loop-Mediated DNA Damage in SMA

Accumulation of R-loops has been reported in several neurological disorders, including ALS, SMA, SMARD1, SCA2, and AOA2 (Figure 3) suggesting that R-loop mediated DNA damage may contribute to the pathogenesis of different genetic diseases. However, the pathogenic mechanisms associated with R-loop accumulation in many of these neurological diseases remain unclear. Nevertheless, some progress has been made towards understanding the cause of R-loop accumulation and the mechanism of R-loop resolution in SMA.

Downregulation of critical factors that may be components of the R-loop resolution complex (RLRC) such as SETX, SMN, and ZPR1, due to SMN-related splicing defects may contribute to R-loop accumulation in SMA (Kannan et al., 2022). Another factor, DEAD/H box protein 9 (DHX9), an RNA helicase, has been shown to contribute to R-loop metabolism but the precise mechanism remains to be examined. DHX9 promotes R-loop accumulation in cells lacking splicing factors and displays defects in pre-mRNA splicing (Chakraborty et al., 2018). DHX9 suppresses defects in RNA processing caused by the Alu-element invasion of the genome (Aktas et al., 2017). Knockdown of DHX9 has been shown cause accumulation of circular RNA (circRNA) for GC- and Alu-element-rich-containing genes, including the *SMN* gene (Ottesen et al., 2019; Shao et al., 2020). Interestingly, circRNA accumulation inhibits DNA repair, which could increase the accumulation of DNA damage caused by R-loops (Xu et al., 2020). This suggests that high levels of circRNA produced by *SMN* genes may be a confounding variable contributing to genomic instability in SMA (Ottesen and Singh, 2020). A recent study identified nucleolar DEAD/H BOX RNA helicase 21 (DDX21) downregulation in SMA patient-derived IPSC motor neurons. DDX21 knockdown results in R-loop accumulation in the nucleolus, which may be a cause of ribosomal DNA damage in SMN-deficient cells (Karyka et al., 2022). Extensive mitochondrial pathology has also been reported in SMA, including mitochondrial DNA (mtDNA) damage, raising the question of whether mtDNA damage in SMA is R-loop mediated (Miller et al., 2016; James et al., 2021). Nevertheless, recent analyses of SMA patient and mouse cells and tissues have provided insight into the mechanism of R-loop-mediated DNA damage and genomic instability in SMA.

Analysis of SMN deficiency was done in non-SMA cells with SMN knockdown (acute) and SMA patient cells (chronic). Acute SMN-deficiency (knockdown) results in DNA damage and activation of DDR pathways in dividing non-SMA SMN-deficient cells. Notably, chronic low levels of SMN protein also result in increased accumulation of DSBs in SMA patient dividing cells as indicated by accumulation of γH2AX and 53BP1 foci compared to control cells. Further analysis showed that the DNA damage caused by either acute or chronic SMN-deficiency was caused by the accumulation of R-loops in dividing non-SMA and SMA patient cells (Kannan et al., 2018). Complementation with ectopic expression of recombinant SMN rescued DNA damage caused by chronic low levels of SMN in SMA patient cells. These findings suggested that the low levels of SMN may also contribute to R-loop accumulation and DNA damage in motor neurons. Further investigation using primary cultured spinal motor neurons derived from SMA mice demonstrated that chronic low levels of SMN indeed resulted in R-loop accumulation, causing DNA damage and leading to the activation of the NHEJ-mediated DNA repair pathway (Kannan et al., 2018). Interestingly, the accumulation of R-loops in SMA motor neurons (non-dividing cells) was higher (~8-fold) compared to (~2-fold) in SMA patient (dividing) cells. This finding suggests an increased accumulation of DNA damage in post-mitotic (non-dividing cells) neurons compared to mitotic cells (Kannan et al., 2018). Higher levels of R-loops in SMA were found to correlate with the downregulation of SETX in SMA patient cells and motor neurons derived from SMA mice. Decreased levels of SETX result in poor efficiency of R-loop resolution and accumulation of DNA damage (Kannan et al., 2018, 2020).

These observations identified SMA as a neurological disorder characterized by R-loop mediated DNA damage and simultaneously opened the door to investigate the consequences of genomic instability on the survival of motor neurons and raised important questions: why were SMA motor neurons showing increased levels of R-loop accumulation as compared to dividing cells? Is an increase in R-loop accumulation making SMA motor neurons “selectively” vulnerable to genomic instability?

## Inefficient DNA Repair Causes Selective Degeneration of Motor Neurons in SMA

SMA is characterized by the selective degeneration of spinal motor neurons (Ahmad et al., 2016). This is not to say that other neurons/non-neural cell types are unaffected by the underlying slow burn of R-loop accumulation and NHEJ pathway downregulation in SMA: but why are spinal motor neurons lost while other cell types survive? SMN is a ubiquitously expressed protein and is essential for viability (Schrank et al., 1997). The question of the specific vulnerability of spinal motor neurons to low levels of the SMN protein in SMA remained enigmatic for researchers in the SMA field for more than two decades (Monani, 2005; Burghes and Beattie, 2009). Recent studies have contributed to understanding the molecular basis of selective degeneration of spinal motor neurons in SMA by providing insight into the impact of impaired DNA repair leading to genomic instability in neurons, but not in dividing patient cells (Kannan et al., 2018, 2020).

As discussed previously, defects in DNA repair result in the accumulation of DNA damage that could lead to genomic instability, therefore, cells must efficiently repair DNA lesions, including DSBs to survive. Experiments involving SMA patient (mitotic) cells and SMA motor neurons (post-mitotic) demonstrated that the HR pathway is intact in mitotic cells but NHEJ pathway is deregulated (Kannan et al., 2018). However, the higher levels of DNA damage in SMA dividing cells compared control cells suggest that HR-mediated DNA repair may also be affected. For example, Gemin2, SMN interacting protein, promotes the accumulation of RAD51 at DSBs and contributes to HR-mediated DNA repair (Takizawa et al., 2010; Takaku et al., 2011). Of note, Gemin2 levels are downregulated in SMA dividing and differentiated cells (Helmken et al., 2003; Wu et al., 2011) suggesting that HR-mediated DNA repair may not be optimal in SMA. SMA patient fibroblasts compensate for higher levels of DNA damage by activating the HR pathway, and to a lesser extent the NHEJ pathway, to repair DNA damage. SMA patient fibroblasts shield themselves from DNA damage using the HR repair pathway during each cell cycle and continue to proliferate. This is consistent with the fact that the bodies of SMA patients continue to grow, while motor neurons unable to sustain growth, innervate rapidly growing skeletal muscle to support normal neuromuscular function (Ahmad et al., 2016; Kannan et al., 2018; Gollapalli et al., 2021). These findings are also consistent with systemic dysfunction of terminally differentiated tissue types such as skeletal muscle, liver, and kidney tissues in SMA patients and mice (Fayzullina and Martin, 2014; Lipnick et al., 2019; Nery et al., 2019; Kim et al., 2020).

**Figure 4:**
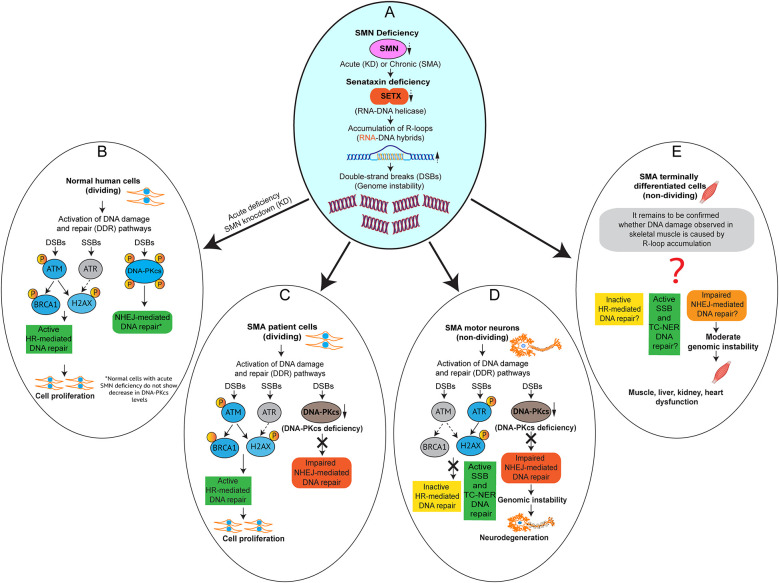
Impaired NHEJ-mediated DNA repair causes genomic instability and selective degeneration of motor neurons in SMA. Graphical models **(A–E)** showing the activation DNA damage response (DDR) pathways caused by acute and chronic SMN-deficiencies, which result in R-loop accumulation leading to DNA damage in SMN-deficient cells. **(A)** SMN deficiency, acute (knockdown of SMN) and chronic (SMA) result in downregulation of SETX, which causes accumulation of R-loops and DNA damage. **(B)** Acute SMN-deficiency in normal human dividing cells causes activation of DDR and Homologous recombination (HR) and non-homologous end joining (NHEJ) DNA repair pathways. **(C)** Chronic SMN-deficiency in SMA patient dividing cells causes activation of DDR and HR to repair double-strand breaks (DSBs) but NHEJ pathway is impaired due to downregulation of DNA-PKcs, a critical factor required for NHEJ-mediated DSBs repair. **(D)** Chronic SMN-deficiency in SMA motor neurons causes activation of DDR. However, HR is inactive and NHEJ pathway is impaired due to downregulation of DNA-PKcs, which results in gradual accumulation of DNA damage leading to genomic instability and motor neuron degeneration. The single-strand breaks (SSBs) and transcription-coupled nucleotide exchange repair (TC-NER) pathways may be active in SMA neurons but not sufficient to prevent DSBs. **(E)** Chronic SMN-deficiency in SMA terminally-differentiated cells other than neurons such as skeletal muscle, liver, kidney, and heart cells may also cause R-loop-mediated DNA damage and activation of SSBs and DSBs repair pathways. Graphical model adapted and modified from Kannan et al. (2018).

Spinal motor neurons bear the bulk of a dual burden in SMA. Importantly, as mentioned above, neurons are high maintenance cells and may be more vulnerable to ROS and transcription-mediated DNA damage due to high rates of oxidative phosphorylation and transcription. In SMA neurons, SMN-deficiency results in the downregulation of SETX causing the accumulation of unresolved co-transcriptional R-loops and leading to increased levels of DSBs. Neurons must rely on the NHEJ pathway for DSBs repair. However, chronic SMN deficiency (unlike acute SMN deficiency, as demonstrated by Kannan et al., 2018), results in the downregulation of DNA-PKcs, a key factor in NHEJ-mediated DNA repair. Thus, the deficiencies of SETX, which results in R-loop accumulation and DNA damage, and DNA-PKcs which partially impairs NHEJ-mediated DNA repair, cause gradual accumulation of irreparable DNA damage that leads to genomic instability and motor neuron degeneration in SMA (Kannan et al., 2018). Overexpression of SMN rescues DNA damage in patient cells and motor neurons from SMA mice by increasing levels of SETX and DNA-PKcs. Of note, ectopic overexpression of SETX in SMA neurons decreases R-loops and rescues DNA damage and prevents degeneration of SMN-deficient motor neurons. These results suggest that pathogenic accumulation of SETX-dependent R-loops may be a major cause of genomic instability and neurodegeneration in SMA (Kannan et al., 2018). These findings raised a hypothesis that SETX might be a protective modifier for the rescue of SMA phenotype and remains to be tested. Notably, these findings establish the importance of NHEJ-mediated DSBs repair in the pathogenesis and the rescue of SMA phenotype in motor neurons. The mechanism of selective degeneration of motor neurons in SMA is illustrated in Figure 4.

## Role of ZPR1 in SMA Pathogenesis and Rescue of R-Loop Mediated DNA Damage

The human *ZPR1*gene is located on Ch11q23.2, is ubiquitously expressed, and is evolutionarily conserved among eukaryotes (Gangwani et al., 1998). ZPR1 binds to inactive receptor kinases (RTKs) and mediates intracellular signaling (Galcheva-Gargova et al., 1996, 1998). ZPR1 is essential for cell viability in yeast and mice (Gangwani et al., 1998, 2005). ZPR1 contains two C4-type zinc fingers with helix-loop-helix motifs that may mediate its interactions with nucleic acids, RNA and DNA, and proteins (Mishra et al., 2007). ZPR1 forms endogenous complexes with SMN and is required for the accumulation of SMN in subnuclear bodies, SMN containing gems and CBs, in the nucleus (Gangwani et al., 2001, 2005). The interaction of ZPR1 and SMN is disrupted in cells derived from SMA patients (Type I) and in the spinal cords from severe SMA mouse models (Gangwani et al., 2001; Ahmad et al., 2012). Reduced *Zpr1* gene dosage results in neurodegeneration and the development of a mild SMA-like phenotype in mice (Doran et al., 2006). Further, the inactivation of *Zpr1* in motor neurons results in phrenic nerve degeneration causing respiratory failure leading to perinatal lethality in mice (Genabai et al., 2017). Poor innervation of diaphragm due to axonal degeneration of ZPR1-deficient phrenic motor neurons results in the loss of synapse and collapse of diaphragmatic rhythm, and respiratory failure. Analysis of SMA mice with chronic low levels of ZPR1 also shows loss of phrenic motor neurons that regulate respiration (Genabai et al., 2017). These observations are in line with clinical reports that show the death of SMA patients is caused by respiratory failure (Ahmad et al., 2016; Genabai et al., 2017). These studies demonstrate that ZPR1 is critical for the growth, maintenance, and survival of the spinal cord motor neurons, including phrenic nerve motor neurons, and suggest that the low levels of ZPR1 observed in SMA patients may contribute to the severity and pathogenesis of SMA.

## Role of ZPR1 in Preventing R-Loop Accumulation and The Rescue of SMA

It is demonstrated that SMN, ZPR1, and SETX interact and form endogenous complexes with RNAPII, and are critical for resolving R-loops. SMN is known to play a role in the assembly of snRNPs and mRNA splicing, SETX in unwinding RNA-DNA helices, and ZPR1 may play a role in regulating pre-mRNA transcription by contributing to the resolution of co-transcriptional R-loops (Gubitz et al., 2004; Bennett and La Spada, 2018; Kannan et al., 2018). The key molecular steps involved in R-loop resolution are unclear, and the role of ZPR1 in regulating transcription remains to be examined. ZPR1 has been known to associate with the SMN 5q13 locus, but whether ZPR1 is a specific trans-acting transcription factor regulating SMN, and has a specific DNA binding site, or has broad specificity for nucleic acids, acting as a part of core transcription complexes and thereby regulating transcription itself remains to be examined (Gangwani, 2006; Kannan et al., 2020). However, in mitotic cells, ZPR1 knockdown is shown to cause defects in transcription and cell cycle progression (Gangwani, 2006). ZPR1 interacts *in vitro* and forms endogenous complexes with RNAPII, and may act as a part of transcription complexes (Kannan et al., 2020). SMN has also been shown to bind to RNAPII and the disruption of RNAPII-SMN complexes causes defects in transcription termination and R-loop accumulation (Zhao et al., 2016). Both RNAPII and SMN are shown to bind to SETX, and these protein complexes may be involved in mRNA biogenesis, including transcription, R-loop resolution, and splicing (Ursic et al., 2004; Suraweera et al., 2009; Yuce and West, 2013). These studies suggest that ZPR1, SMN, SETX, and RNAPII collaborate and contribute to the transcription and resolution of nascent pre-mRNA from the transcribing DNA strand, resulting in the resolution of co-transcriptional R-loops. However, the precise role/contribution of each protein in mRNA biogenesis, specifically in R-loop resolution, remains to be studied.

Some progress has been made to uncover the role of ZPR1 in R-loop metabolism. A recent study demonstrated that the knockdown of ZPR1 in mammalian cells results in the accumulation of R-loops and DNA damage as indicated by the formation of γH2AX and 53BP1 foci in ZPR1-deficient cells suggesting that ZPR1-dependent R-loop accumulation induces DNA damage (Kannan et al., 2020). This is consistent with the finding that SMA patient cells have low levels of ZPR1 (Gangwani et al., 2001), and accumulate R-loops and DNA damage at higher rates than normal cells (Kannan et al., 2020). Interestingly, ZPR1 has been shown to be a protective modifier in SMA, reducing R-loops and ameliorating disease phenotype in a severe SMA mouse model (Kannan et al., 2020). There are indications that ZPR1 may function upstream of SMN, which is downregulated in response to ZPR1 knockdown, and SMN levels are elevated in SMA mice and SMA patient cells in response to ZPR1 upregulation (Ahmad et al., 2012; Kannan et al., 2020). In addition to SMN, ZPR1 overexpression upregulated levels of SETX and DNA-PKcs in the CNS of SMA mice. An increase in SETX levels correlates with a decrease in R-loop levels and an increase in DNA-PKcs correlates with reduced DNA damage as indicated by the decrease in γH2AX levels. Basically, ZPR1 provides a two-fold protection and rescues DNA damage by: (i) decreasing R-loop levels and (Orii et al., 2006) increasing the efficiency of DNA-PKcs-dependent NHEJ-mediated DNA repair (Kannan et al., 2020). Further, the overexpression of ZPR1 in SMA mice (Z-SMA) shows a rescue of disease phenotype in mice with severe SMA, increasing survival of Z-SMA mice ~3.5-fold compared to littermates without ZPR1 (SMA; Kannan et al., 2020). These findings suggest that: (i) ZPR1 deficiency contributes to SMA pathogenesis *via* R-loop-mediated DNA damage leading to genomic instability and neurodegeneration; and (ii) ZPR1 rescues DNA damage by preventing the accumulation of pathogenic R-loops and may act as a protective modifier of SMA (Kannan et al., 2020; Cuartas and Gangwani, 2022).

## Mechanism of R-Loop Resolution

Several protein factors, SETX, DHX9, ZPR1, and XRN2 have been identified that may contribute to R-loop metabolism (Bhatia et al., 2017; Cristini et al., 2018; Wang et al., 2018; Kannan et al., 2020). One of the important proteins is SETX, an RNA/DNA helicase that unwinds RNA:DNA hybrids and helps resolve R-loops (Skourti-Stathaki et al., 2011; Cohen et al., 2018; Rawal et al., 2020). The unwinding of RNA:DNA hybrids by SETX may be one of the key steps in the separation of RNA from DNA. How specific factors may contribute to the separation of newly synthesized RNA from DNA and the resolution of R-loops remains unclear.

A recently published work has helped clarify the molecular mechanism of R-loop resolution and provided insight into the putative key steps of R-loop resolution (Kannan et al., 2022). This study utilized two patient cell-based model systems: (i) SMA with higher levels of R-loops; and (ii) ALS4 with lower levels of R-loops compared to normal human cells. Briefly, the findings of this investigation show that SETX interacts *in vitro* and *in vivo* with ZPR1. Notably, ZPR1 binds to R-loops and is required for the recruitment of SETX onto R-loops. The downregulation of SETX and ZPR1 proteins in SMA is due to reduced mRNA expression of SETX and ZPR1 because of likely defects in splicing caused by chronic SMN-deficiency in SMA (Helmken et al., 2003; Kannan et al., 2020). The low levels of SETX-ZPR1 complexes correlate with reduced assembly of the RLRC resulting in inefficient resolution of R-loops leading to the accumulation of higher level of R-loops in SMA compared to control (Kannan et al., 2022).

**Figure 5:**
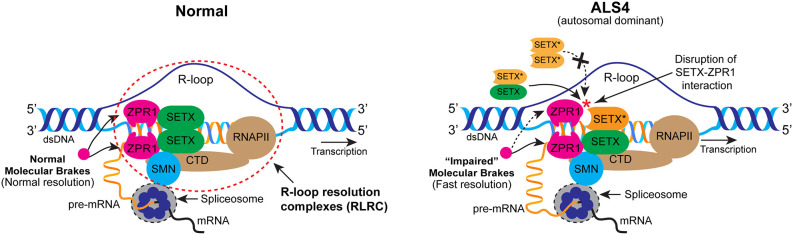
Mechanism of R-loop resolution under the normal and ALS4 disease conditions. In normal cells, ZPR1 binds RNA Pol II (RNAPII) COOH-terminal (CTD) domain. ZPR1 binds to SETX and recruits SETX onto R-loops. ZPR1 tethers to RNA:DNA hybrids and may function as a “molecular brake” to regulate the speed of SETX-dependent R-loop resolution. Nascent RNA hybridized to DNA is separated by the activity of R-loop-resolution complexes (RLRC; dotted ellipse) to make pre-mRNA available for processing and splicing. In ALS4, mutation in SETX (L389S) disrupts its interaction with ZPR1. However, mutation does not affect SETX dimerization. ZPR1 fails to recruit mutant SETX* (orange) homodimer but recruits SETX-SETX* (green - orange) heterodimer onto R-loops. Recruitment of heterodimer results in the partial impairment of the molecular brake and increase in the speed of SETX-dependent R-loop resolution leading to gain-of-function and fewer R-loops in patient cells.

Interestingly, the mutation in SETX (L389S) that causes autosomal dominant ALS4, results in disruption of SETX-ZPR1 interaction and lower levels of R-loops (Chen et al., 2004; Kannan et al., 2022). The recruitment of SETX by ZPR1 onto R-loops was markedly reduced in ALS4 resulting in the low levels of SETX-ZPR1 complexes in RLRCs. The heterozygous L389S mutation results in 50% mutant SETX, which ZPR1 fails to recruit onto R-loops in ALS4. Notably, the expression levels of SETX, ZPR1, and SMN proteins and mRNA were not altered in cells derived from ALS4 patients, suggesting that ZPR1 was unable to recruit mutant SETX onto R-loops. Another notable aspect of these findings is that the ability of SETX to form dimers was not affected by the L389S mutation (Bennett et al., 2013). This suggests that ZPR1 may be able to recruit heterodimer of SETX wild-type and mutant proteins (SETX-SETX^L389S^). The analysis of R-loop resolution complexes (RLRCs) shows reduced levels of ZPR1 and SETX on R-loops in ALS4 cells, a similar situation to SMA. The low levels of SETX-ZPR1 complexes in SMA are caused by the downregulation of these proteins and result in R-loop accumulation. In contrast, ALS4 patient cells show lower levels of R-loops compared to normal cells, supporting the idea of gain-of-function in R-loop resolution, which aligns with the characteristics of an autosomal dominant ALS4 (Kannan et al., 2022). In summary, findings from this study suggest that SETX-ZPR1 complexes are critical for R-loop resolution and ZPR1 may regulate the activity of SETX. The disruption of SETX-ZPR1 complexes may be a cause of gain-of-function because ZPR1 may function as a molecular brake and regulate SETX-dependent R-loop resolution activity, and the impairment of the molecular brake may result in increased speed of R-loop resolution in ALS4 (Figure 5).

Identification of ZPR1 as a molecular factor with the potential to modulate R-loop levels under normal and disease (ALS and SMA) conditions, raises an attractive hypothesis: ZPR1 may be a critical player in guarding the integrity of the genome by regulating R-loop homeostasis. It is possible that ZPR1 deregulation or disruption of ZPR1 protein-protein complexes may be a common contributing factor in the pathogenesis of neurodegenerative diseases caused by R-loop-mediated genomic instability but remains to be investigated.

Together, recent findings open a new chapter in understanding the roles that R-loop mediated DNA damage and impaired DNA repair play in the pathogenesis of neurological disorders. Several studies, including the effect of ALS causing mutations on alteration of SMN function, loss of SMN containing gems in ALS patient cells, and the disruption of common protein networks in SMA and ALS have pointed to overlaps in the patho-mechanisms associated with two genetic neuromuscular disorders, ALS and SMA (Veldink et al., 2005; Achsel et al., 2013; Cauchi, 2014; Sun et al., 2015; Kubinski and Claus, 2022). One of the common links between different neurodegenerative disorders highlighted here is the gradual accumulation of DNA damage leading to genomic instability as one of the hallmarks of neurodegeneration. To parse out the common molecular mechanisms that are involved in genomic instability-mediated neurodegeneration, further research that utilizes multiple neurodegenerative disease models will be enlightening.

## Author Contributions

JC and LG conceived the topic of review and edited the manuscript and figure. JC prepared the draft of manuscript and figures. All authors contributed to the article and approved the submitted version.

## Conflict of Interest

The authors declare that the research was conducted in the absence of any commercial or financial relationships that could be construed as a potential conflict of interest.

## Publisher’s Note

All claims expressed in this article are solely those of the authors and do not necessarily represent those of their affiliated organizations, or those of the publisher, the editors and the reviewers. Any product that may be evaluated in this article, or claim that may be made by its manufacturer, is not guaranteed or endorsed by the publisher.

## References

[B1] Achsel T. Barabino S. Cozzolino M. Carri M. T. (2013). The intriguing case of motor neuron disease: ALS and SMA come closer. Biochem. Soc. Trans. 41, 1593–1597. 10.1042/BST20130142 24256260

[B2] Ahmad S. Bhatia K. Kannan A. Gangwani L. (2016). Molecular mechanisms of neurodegeneration in spinal muscular atrophy. J. Exp. Neurosci. 10, 39–49. 10.4137/JEN.S33122 27042141 PMC4807884

[B3] Ahmad S. Wang Y. Shaik G. M. Burghes A. H. Gangwani L. (2012). The zinc finger protein ZPR1 is a potential modifier of spinal muscular atrophy. Human Mol. Genet. 21, 2745–2758. 10.1093/hmg/dds102 22422766 PMC3363332

[B4] Aktas T. Avsar Ilik I. Maticzka D. Bhardwaj V. Pessoa Rodrigues C. Mittler G.. (2017). DHX9 suppresses RNA processing defects originating from the Alu invasion of the human genome. Nature 544, 115–119. 10.1038/nature21715 28355180

[B5] Barnes D. E. Stamp G. Rosewell I. Denzel A. Lindahl T. (1998). Targeted disruption of the gene encoding DNA ligase IV leads to lethality in embryonic mice. Curr. Biol. 8, 1395–1398. 10.1016/s0960-9822(98)00021-9 9889105

[B6] Barzilai A. (2007). The contribution of the DNA damage response to neuronal viability. Antioxid. Redox Signal. 9, 211–218. 10.1089/ars.2007.9.211 17115940

[B7] Barzilai A. Biton S. Shiloh Y. (2008). The role of the DNA damage response in neuronal development, organization and maintenance. DNA Repair (Amst) 7, 1010–1027. 10.1016/j.dnarep.2008.03.005 18458000

[B8] Bayat V. Jaiswal M. Bellen H. J. (2011). The BMP signaling pathway at the *Drosophila* neuromuscular junction and its links to neurodegenerative diseases. Curr. Opin. Neurobiol. 21, 182–188. 10.1016/j.conb.2010.08.014 20832291 PMC3095363

[B9] Becherel O. J. Sun J. Yeo A. J. Nayler S. Fogel B. L. Gao F.. (2015). A new model to study neurodegeneration in ataxia oculomotor apraxia type 2. Hum. Mol. Genet. 24, 5759–5774. 10.1093/hmg/ddv296 26231220 PMC4581605

[B11] Bennett C. L. Chen Y. Vignali M. Lo R. S. Mason A. G. Unal A.. (2013). Protein interaction analysis of senataxin and the ALS4 L389S mutant yields insights into senataxin post-translational modification and uncovers mutant-specific binding with a brain cytoplasmic RNA-encoded peptide. PLoS One 8:e78837. 10.1371/journal.pone.0078837 24244371 PMC3823977

[B12] Bennett C. L. Dastidar S. G. Ling S. C. Malik B. Ashe T. Wadhwa M.. (2018). Senataxin mutations elicit motor neuron degeneration phenotypes and yield TDP-43 mislocalization in ALS4 mice and human patients. Acta Neuropathol. 136, 425–443. 10.1007/s00401-018-1852-9 29725819 PMC6098723

[B10] Bennett C. L. La Spada A. R. (2018). Senataxin, a novel helicase at the interface of RNA transcriptome regulation and neurobiology: from normal function to pathological roles in motor neuron disease and cerebellar degeneration. Adv. Neurobiol. 20, 265–281. 10.1007/978-3-319-89689-2_10 29916023

[B13] Bhatia V. Herrera-Moyano E. Aguilera A. Gomez-Gonzalez B. (2017). The role of replication-associated repair factors on R-loops. Genes (Basel) 8:171. 10.3390/genes8070171 28653981 PMC5541304

[B14] Bowerman M. Shafey D. Kothary R. (2007). Smn depletion alters profilin II expression and leads to upregulation of the RhoA/ROCK pathway and defects in neuronal integrity. J. Mol. Neurosci. 32, 120–131. 10.1007/s12031-007-0024-5 17873296

[B15] Branzei D. Foiani M. (2008). Regulation of DNA repair throughout the cell cycle. Nat. Rev. Mol. Cell Biol. 9, 297–308. 10.1038/nrm2351 18285803

[B16] Burghes A. H. Beattie C. E. (2009). Spinal muscular atrophy: why do low levels of survival motor neuron protein make motor neurons sick? Nat. Rev. Neurosci. 10, 597–609. 10.1038/nrn2670 19584893 PMC2853768

[B17] Cauchi R. J. (2014). Gem depletion: amyotrophic lateral sclerosis and spinal muscular atrophy crossover. CNS Neurosci. Ther. 20, 574–581. 10.1111/cns.12242 24645792 PMC6492989

[B18] Chakraborty P. Huang J. T. J. Hiom K. (2018). DHX9 helicase promotes R-loop formation in cells with impaired RNA splicing. Nat. Commun. 9:4346. 10.1038/s41467-018-06677-1 30341290 PMC6195550

[B19] Chang H. C. Dimlich D. N. Yokokura T. Mukherjee A. Kankel M. W. Sen A.. (2008). Modeling spinal muscular atrophy in *Drosophila*. PLoS One 3:e3209. 10.1371/journal.pone.0003209 18791638 PMC2527655

[B20] Chao C. Saito S. Anderson C. W. Appella E. Xu Y. (2000). Phosphorylation of murine p53 at ser-18 regulates the p53 responses to DNA damage. Proc. Natl. Acad. Sci. U S A 97, 11936–11941. 10.1073/pnas.220252297 11035798 PMC17273

[B21] Chatterjee N. Walker G. C. (2017). Mechanisms of DNA damage, repair and mutagenesis. Environ. Mol. Mutagen. 58, 235–263. 10.1002/em.22087 28485537 PMC5474181

[B22] Chen Y. Z. Bennett C. L. Huynh H. M. Blair I. P. Puls I. Irobi J.. (2004). DNA/RNA helicase gene mutations in a form of juvenile amyotrophic lateral sclerosis (ALS4). Am. J. Hum. Genet. 74, 1128–1135. 10.1086/421054 15106121 PMC1182077

[B23] Cheng C. Seen D. Zheng C. Zeng R. Li E. (2021). Role of small GTPase RhoA in DNA damage response. Biomolecules 11:212. 10.3390/biom11020212 33546351 PMC7913530

[B24] Cohen S. Puget N. Lin Y. L. Clouaire T. Aguirrebengoa M. Rocher V.. (2018). Senataxin resolves RNA:DNA hybrids forming at DNA double-strand breaks to prevent translocations. Nat. Commun. 9:533. 10.1038/s41467-018-02894-w 29416069 PMC5803260

[B25] Cristini A. Groh M. Kristiansen M. S. Gromak N. (2018). RNA/DNA hybrid interactome identifies dxh9 as a molecular player in transcriptional termination and R-loop-associated DNA damage. Cell Rep. 23, 1891–1905. 10.1016/j.celrep.2018.04.025 29742442 PMC5976580

[B26] Cristini A. Ricci G. Britton S. Salimbeni S. Huang S. Y. N. Marinello J.. (2019). Dual processing of R-loops and topoisomerase I induces transcription-dependent DNA double-strand breaks. Cell Rep. 28:3167. 10.1016/j.celrep.2019.08.041 31533039 PMC8274950

[B500] Crossley M. P. Bocek M. Cimprich K. A. (2019). R-Loops as cellular regulators and genomic threats. Mol. Cell 73, 398–411. 10.1016/j.molcel.2019.01.024 30735654 PMC6402819

[B27] Cuartas J. Gangwani L. (2022). Zinc finger protein ZPR1: promising survival motor neuron protein-dependent modifier for the rescue of spinal muscular atrophy. Neural Regen. Res. 17, 2225–2227. 10.4103/1673-5374.335798 35259840 PMC9083177

[B28] D’Alessandro G. d’Adda Di Fagagna F. (2017). Transcription and DNA damage: holding hands or crossing swords? J. Mol. Biol. 429, 3215–3229. 10.1016/j.jmb.2016.11.002 27825959

[B29] Doran B. Gherbesi N. Hendricks G. Flavell R. A. Davis R. J. Gangwani L.. (2006). Deficiency of the zinc finger protein ZPR1 causes neurodegeneration. Proc. Natl. Acad. Sci. U S A 103, 7471–7475. 10.1073/pnas.0602057103 16648254 PMC1464363

[B30] Fayzullina S. Martin L. J. (2014). Skeletal muscle DNA damage precedes spinal motor neuron DNA damage in a mouse model of spinal muscular atrophy (SMA). PLoS One 9:e93329. 10.1371/journal.pone.0093329 24667816 PMC3965546

[B31] Fayzullina S. Martin L. J. (2016). DNA damage response and DNA repair in skeletal myocytes from a mouse model of spinal muscular atrophy. J. Neuropathol. Exp. Neurol. 75, 889–902. 10.1093/jnen/nlw064 27452406 PMC5015659

[B32] Fogel B. L. Cho E. Wahnich A. Gao F. Becherel O. J. Wang X.. (2014). Mutation of senataxin alters disease-specific transcriptional networks in patients with ataxia with oculomotor apraxia type 2. Hum. Mol. Genet. 23, 4758–4769. 10.1093/hmg/ddu190 24760770 PMC4140459

[B33] Frank K. M. Sharpless N. E. Gao Y. Sekiguchi J. M. Ferguson D. O. Zhu C.. (2000). DNA ligase IV deficiency in mice leads to defective neurogenesis and embryonic lethality via the p53 pathway. Mol. Cell 5, 993–1002. 10.1016/s1097-2765(00)80264-6 10911993

[B34] Fuchs S. Y. Adler V. Pincus M. R. Ronai Z. (1998). MEKK1/JNK signaling stabilizes and activates p53. Proc. Natl. Acad. Sci. U S A 95, 10541–10546. 10.1073/pnas.95.18.10541 9724739 PMC27930

[B35] Galcheva-Gargova Z. Gangwani L. Konstantinov K. N. Mikrut M. Theroux S. J. Enoch T.. (1998). The cytoplasmic zinc finger protein ZPR1 accumulates in the nucleolus of proliferating. Mol. Biol. Cell 9, 2963–2971. 10.1091/mbc.9.10.2963 9763455 PMC25573

[B36] Galcheva-Gargova Z. Konstantinov K. N. Wu I. H. Klier F. G. Barrett T. Davis R. J.. (1996). Binding of zinc finger protein ZPR1 to the epidermal growth factor receptor. Science 272, 1797–1802. 10.1126/science.272.5269.1797 8650580

[B37] Gangwani L. (2006). Deficiency of the zinc finger protein ZPR1 causes defects in transcription and cell cycle progression. J. Biol. Chem. 281, 40330–40340. 10.1074/jbc.M608165200 17068332

[B38] Gangwani L. Flavell R. A. Davis R. J. (2005). ZPR1 is essential for survival and is required for localization of the survival motor neurons (SMN) protein to Cajal bodies. Mol. Cell. Biol. 25, 2744–2756. 10.1128/MCB.25.7.2744-2756.2005 15767679 PMC1061650

[B39] Gangwani L. Mikrut M. Galcheva-Gargova Z. Davis R. J. (1998). Interaction of ZPR1 with translation elongation factor-1alpha in proliferating cells. J. Cell. Biol. 143, 1471–1484. 10.1083/jcb.143.6.1471 9852145 PMC2132977

[B40] Gangwani L. Mikrut M. Theroux S. Sharma M. Davis R. J. (2001). Spinal muscular atrophy disrupts the interaction of ZPR1 with the SMN protein. Nat. Cell Biol. 3, 376–383. 10.1038/35070059 11283611

[B41] Garcia-Muse T. Aguilera A. (2019). R loops: from physiological to pathological roles. Cell 179, 604–618. 10.1016/j.cell.2019.08.055 31607512

[B42] Genabai N. K. Ahmad S. Zhang Z. Jiang X. Gabaldon C. A. Gangwani L.. (2015). Genetic inhibition of JNK3 ameliorates spinal muscular atrophy. Hum. Mol. Genet. 24, 6986–7004. 10.1093/hmg/ddv401 26423457 PMC4654054

[B43] Genabai N. K. Kannan A. Ahmad S. Jiang X. Bhatia K. Gangwani L.. (2017). Deregulation of ZPR1 causes respiratory failure in spinal muscular atrophy. Sci. Rep. 7:8295. 10.1038/s41598-017-07603-z 28811488 PMC5557895

[B44] Giannini M. Bayona-Feliu A. Sproviero D. Barroso S. I. Cereda C. Aguilera A.. (2020). TDP-43 mutations link amyotrophic lateral sclerosis with R-loop homeostasis and R loop-mediated DNA damage. PLoS Genet. 16:e1009260. 10.1371/journal.pgen.1009260 33301444 PMC7755276

[B45] Gollapalli K. Kim J. K. Monani U. R. (2021). Emerging concepts underlying selective neuromuscular dysfunction in infantile-onset spinal muscular atrophy. Neural Regen. Res. 16, 1978–1984. 10.4103/1673-5374.308073 33642371 PMC8343306

[B46] Groh M. Gromak N. (2014). Out of balance: R-loops in human disease. PLoS Genet. 10:e1004630. 10.1371/journal.pgen.1004630 25233079 PMC4169248

[B47] Grunseich C. Wang I. X. Watts J. A. Burdick J. T. Guber R. D. Zhu Z.. (2018). Senataxin mutation reveals how R-loops promote transcription by blocking DNA methylation at gene promoters. Mol. Cell 69, 426–437.e7. 10.1016/j.molcel.2017.12.030 29395064 PMC5815878

[B48] Gubitz A. K. Feng W. Dreyfuss G. (2004). The SMN complex. Exp. Cell Res. 296, 51–56. 10.1016/j.yexcr.2004.03.022 15120993

[B49] Hamperl S. Bocek M. J. Saldivar J. C. Swigut T. Cimprich K. A. (2017). Transcription-replication conflict orientation modulates R-loop levels and activates distinct DNA damage responses. Cell 170:774. 10.1016/j.cell.2017.07.043 28802045 PMC5570545

[B50] Hegazy Y. A. Fernando C. M. Tran E. J. (2020). The balancing act of R-loop biology: the good, the bad and the ugly. J. Biol. Chem. 295, 905–913. 10.1074/jbc.REV119.011353 31843970 PMC6983857

[B51] Hegde M. L. Bohr V. A. Mitra S. (2017). DNA damage responses in central nervous system and age-associated neurodegeneration. Mech. Ageing Dev. 161, 1–3. 10.1016/j.mad.2017.01.010 28212866 PMC5458627

[B52] Helmken C. Hofmann Y. Schoenen F. Oprea G. Raschke H. Rudnik-Schoneborn S.. (2003). Evidence for a modifying pathway in SMA discordant families: reduced SMN level decreases the amount of its interacting partners and Htra2-beta1. Hum. Genet. 114, 11–21. 10.1007/s00439-003-1025-2 14520560

[B53] Hensel N. Detering N. T. Walter L. M. Claus P. (2020). Resolution of pathogenic R-loops rescues motor neuron degeneration in spinal muscular atrophy. Brain 143, 2–5. 10.1093/brain/awz394 31886489

[B54] James R. Chaytow H. Ledahawsky L. M. Gillingwater T. H. (2021). Revisiting the role of mitochondria in spinal muscular atrophy. Cell. Mol. Life Sci. 78, 4785–4804. 10.1007/s00018-021-03819-5 33821292 PMC8195803

[B55] Jangi M. Fleet C. Cullen P. Gupta S. V. Mekhoubad S. Chiao E.. (2017). SMN deficiency in severe models of spinal muscular atrophy causes widespread intron retention and DNA damage. Proc. Natl. Acad. Sci. U S A 114, E2347–E2356. 10.1073/pnas.1613181114 28270613 PMC5373344

[B56] Jiang X. Kannan A. Gangwani L. (2019). ZPR1-dependent neurodegeneration is mediated by the JNK signaling pathway. J. Exp. Neurosci. 13:1179069519867915. 10.1177/1179069519867915 31488953 PMC6709431

[B57] Kannan A. Bhatia K. Branzei D. Gangwani L. (2018). Combined deficiency of Senataxin and DNA-PKcs causes DNA damage accumulation and neurodegeneration in spinal muscular atrophy. Nucleic Acids Res. 46, 8326–8346. 10.1093/nar/gky641 30010942 PMC6144794

[B58] Kannan A. Cuartas J. Gangwani P. Branzei D. Gangwani L. (2022). Mutation in senataxin alters the mechanism of R-loop resolution in amyotrophic lateral sclerosis 4. Brain 19:awab464. 10.1093/brain/awab464 35045161 PMC9536298

[B59] Kannan A. Jiang X. He L. Ahmad S. Gangwani L. (2020). ZPRI prevents R-loop accumulation, upregulates SMN2 expression and rescues spinal muscular atrophy. Brain 143, 69–93. 10.1093/brain/awz373 31828288 PMC6935747

[B60] Kanungo J. (2013). DNA-dependent protein kinase and DNA repair: relevance to Alzheimer’s disease. Alzheimers Res. Ther. 5:13. 10.1186/alzrt167 23566654 PMC3706827

[B61] Kanungo J. (2016). DNA-PK Deficiency in Alzheimer’s disease. J. Neurol. Neuromedicine 1, 17–22. 10.29245/2572.942x/2016/3.1016 27376156 PMC4924576

[B62] Karyka E. Berrueta Ramirez N. Webster C. P. Marchi P. M. Graves E. J. Godena V. K.. (2022). SMN-deficient cells exhibit increased ribosomal DNA damage. Life Sci. Alliance 5:e202101145. 10.26508/lsa.202101145 35440492 PMC9018017

[B63] Kastan M. B. (2008). DNA damage responses: mechanisms and roles in human disease: 2007 G.H.A. Clowes Memorial Award Lecture. Mol. Cancer Res. 6, 517–524. 10.1158/1541-7786.MCR-08-0020 18403632

[B64] Kim J. K. Jha N. N. Feng Z. Faleiro M. R. Chiriboga C. A. Wei-Lapierre L.. (2020). Muscle-specific SMN reduction reveals motor neuron-independent disease in spinal muscular atrophy models. J. Clin. Invest. 130, 1271–1287. 10.1172/JCI131989 32039917 PMC7269591

[B65] Konopka A. Whelan D. R. Jamali M. S. Perri E. Shahheydari H. Toth R. P.. (2020). Impaired NHEJ repair in amyotrophic lateral sclerosis is associated with TDP-43 mutations. Mol. Neurodegener. 15:51. 10.1186/s13024-020-00386-4 32907630 PMC7488163

[B66] Kubinski S. Claus P. (2022). Protein network analysis reveals a functional connectivity of dysregulated processes in ALS and SMA. Neurosci. Insights 17:26331055221087740. 10.1177/26331055221087740 35372839 PMC8966079

[B67] Lans H. Hoeijmakers J. H. J. Vermeulen W. Marteijn J. A. (2019). The DNA damage response to transcription stress. Nat. Rev. Mol. Cell Biol. 20, 766–784. 10.1038/s41580-019-0169-4 31558824

[B68] Li X. Manley J. L. (2005). Inactivation of the SR protein splicing factor ASF/SF2 results in genomic instability. Cell 122, 365–378. 10.1016/j.cell.2005.06.008 16096057

[B69] Lin X. Kapoor A. Gu Y. Chow M.J. Peng J. Zhao K.. (2020). Contributions of DNA damage to Alzheimer’s disease. Int. J. Mol. Sci. 21:1666. 10.3390/ijms21051666 32121304 PMC7084447

[B70] Lin Y. Wilson J. H. (2012). Nucleotide excision repair, mismatch repair and R-loops modulate convergent transcription-induced cell death and repeat instability. PLoS One 7:e46807. 10.1371/journal.pone.0046807 23056461 PMC3463551

[B71] Lipnick S. L. Agniel D. M. Aggarwal R. Makhortova N. R. Finlayson S. G. Brocato A.. (2019). Systemic nature of spinal muscular atrophy revealed by studying insurance claims. PLoS One 14:e0213680. 10.1371/journal.pone.0213680 30870495 PMC6417721

[B72] Lorson C. L. Hahnen E. Androphy E. J. Wirth B. (1999). A single nucleotide in the SMN gene regulates splicing and is responsible for spinal muscular atrophy. Proc. Natl. Acad. Sci. U S A 96, 6307–6311. 10.1073/pnas.96.11.6307 10339583 PMC26877

[B73] Lu W. T. Hawley B. R. Skalka G. L. Baldock R. A. Smith E. M. Bader A. S.. (2018). Drosha drives the formation of DNA:RNA hybrids around DNA break sites to facilitate DNA repair. Nat. Commun. 9:532. 10.1038/s41467-018-02893-x 29416038 PMC5803274

[B74] Mackay R. P. Xu Q. Weinberger P. M. (2020). R-loop physiology and pathology: a brief review. DNA Cell. Biol. 39, 1914–1925. 10.1089/dna.2020.5906 33052725

[B75] Madabhushi R. Pan L. Tsai L. H. (2014). DNA damage and its links to neurodegeneration. Neuron 83, 266–282. 10.1016/j.neuron.2014.06.034 25033177 PMC5564444

[B76] Magalhaes Y. T. Farias J. O. Silva L. E. Forti F. L. (2021). GTPases, genome, actin: a hidden story in DNA damage response and repair mechanisms. DNA Repair (Amst) 100:103070. 10.1016/j.dnarep.2021.103070 33618126

[B501] Mao Z. Bozzella M. Seluanov A. Gorbunova V. (2008). DNA repair by nonhomologous end joining and homologous recombination during cell cycle in human cells. Cell Cycle 7, 2902–2906. 10.4161/cc.7.18.6679 18769152 PMC2754209

[B77] Markowitz J. A. Singh P. Darras B. T. (2012). Spinal muscular atrophy: a clinical and research update. Pediatr. Neurol. 46, 1–12. 10.1016/j.pediatrneurol.2011.09.001 22196485

[B78] Marnef A. Legube G. (2021). R-loops as Janus-faced modulators of DNA repair. Nat. Cell Biol. 23, 305–313. 10.1038/s41556-021-00663-4 33837288

[B79] McKinnon P. J. (2009). DNA repair deficiency and neurological disease. Nat. Rev. Neurosci. 10, 100–112. 10.1038/nrn2559 19145234 PMC3064843

[B80] Miller N. Shi H. Zelikovich A. S. Ma Y. C. (2016). Motor neuron mitochondrial dysfunction in spinal muscular atrophy. Hum. Mol. Genet. 25, 3395–3406. 10.1093/hmg/ddw262 27488123 PMC5179954

[B81] Mishra A. K. Gangwani L. Davis R. J. Lambright D. G. (2007). Structural insights into the interaction of the evolutionarily conserved ZPR1 domain tandem with eukaryotic EF1A, receptors and SMN complexes. Proc. Natl. Acad. Sci. U S A 104, 13930–13935. 10.1073/pnas.0704915104 17704259 PMC1955815

[B82] Mitra J. Guerrero E. N. Hegde P. M. Liachko N. F. Wang H. B. Vasquez V.. (2019). Motor neuron disease-associated loss of nuclear TDP-43 is linked to DNA double-strand break repair defects. Proc. Natl. Acad. Sci. U S A 116, 4696–4705. 10.1073/pnas.1818415116 30770445 PMC6410842

[B83] Monani U. R. (2005). Spinal muscular atrophy: a deficiency in a ubiquitous protein; a motor neuron-specific disease. Neuron 48, 885–896. 10.1016/j.neuron.2005.12.001 16364894

[B84] Nakazawa Y. Hara Y. Oka Y. Komine O. Van Den Heuvel D. Guo C.. (2020). Ubiquitination of DNA damage-stalled RNAPII promotes transcription-coupled repair. Cell 180, 1228–1244.e24. 10.1016/j.cell.2020.02.010 32142649

[B85] Nery F. C. Siranosian J. J. Rosales I. Deguise M. O. Sharma A. Muhtaseb A. W.. (2019). Impaired kidney structure and function in spinal muscular atrophy. Neurol. Genet. 5:e353. 10.1212/NXG.0000000000000353 31517062 PMC6705648

[B86] Niehrs C. Luke B. (2020). Regulatory R-loops as facilitators of gene expression and genome stability. Nat. Rev. Mol. Cell Biol. 21, 167–178. 10.1038/s41580-019-0206-3 32005969 PMC7116639

[B87] Nolle A. Zeug A. Van Bergeijk J. Tonges L. Gerhard R. Brinkmann H.. (2011). The spinal muscular atrophy disease protein SMN is linked to the Rho-kinase pathway via profilin. Hum. Mol. Genet. 20, 4865–4878. 10.1093/hmg/ddr425 21920940

[B88] O’Driscoll M. Jeggo P. A. (2008). The role of the DNA damage response pathways in brain development and microcephaly: insight from human disorders. DNA Repair (Amst) 7, 1039–1050. 10.1016/j.dnarep.2008.03.018 18458003

[B89] Ohle C. Tesorero R. Schermann G. Dobrev N. Sinning I. Fischer T.. (2016). Transient RNA-DNA hybrids are required for efficient double-strand break repair. Cell 167, 1001–1013.e7. 10.1016/j.cell.2016.10.001 27881299

[B90] Okamoto Y. Abe M. Itaya A. Tomida J. Ishiai M. Takaori-Kondo A.. (2019a). FANCD2 protects genome stability by recruiting RNA processing enzymes to resolve R-loops during mild replication stress. FEBS J. 286, 139–150. 10.1111/febs.14700 30431240

[B91] Okamoto Y. Hejna J. Takata M. (2019b). Regulation of R-loops and genome instability in Fanconi anemia. J. Biochem. 165, 465–470. 10.1093/jb/mvz019 30821334

[B92] Orii K. E. Lee Y. Kondo N. Mckinnon P. J. (2006). Selective utilization of nonhomologous end-joining and homologous recombination DNA repair pathways during nervous system development. Proc. Natl. Acad. Sci. U S A 103, 10017–10022. 10.1073/pnas.0602436103 16777961 PMC1502498

[B94] Ottesen E. W. Luo D. Seo J. Singh N. N. Singh R. N. (2019). Human Survival Motor Neuron genes generate a vast repertoire of circular RNAs. Nucleic Acids Res. 47, 2884–2905. 10.1093/nar/gkz034 30698797 PMC6451121

[B93] Ottesen E. W. Singh R. N. (2020). Characteristics of circular RNAs generated by human survival motor neuron genes. Cell Signal. 73:109696. 10.1016/j.cellsig.2020.109696 32553550 PMC7387165

[B95] Perego M. G. L. Taiana M. Bresolin N. Comi G. P. Corti S. (2019). R-loops in motor neuron diseases. Mol. Neurobiol. 56, 2579–2589. 10.1007/s12035-018-1246-y 30047099

[B96] Picco V. Pages G. (2013). Linking JNK activity to the DNA damage response. Genes Cancer 4, 360–368. 10.1177/1947601913486347 24349633 PMC3863338

[B98] Rao K. S. (2007). DNA repair in aging rat neurons. Neuroscience 145, 1330–1340. 10.1016/j.neuroscience.2006.09.032 17156934

[B97] Rao C. V. Farooqui M. Zhang Y. T. Asch A. S. Yamada H. Y. (2018). Spontaneous development of Alzheimer’s disease-associated brain pathology in a Shugoshin-1 mouse cohesinopathy model. Aging Cell 17:e12797. 10.1111/acel.12797 29943428 PMC6052391

[B99] Rass U. Ahel I. West S. C. (2007). Defective DNA repair and Neurodegenerative disease. Cell 130, 991–1004. 10.1016/j.cell.2007.08.043 17889645

[B100] Rawal C. C. Zardoni L. Di Terlizzi M. Galati E. Brambati A. Lazzaro F.. (2020). Senataxin ortholog sen1 limits DNA:RNA hybrid accumulation at DNA double-strand breaks to control end resection and repair fidelity. Cell Rep. 31:107603. 10.1016/j.celrep.2020.107603 32375052

[B101] Richard P. Manley J. L. (2017). R loops and links to human disease. J. Mol. Biol. 429, 3168–3180. 10.1016/j.jmb.2016.08.031 27600412 PMC5478472

[B102] Saini N. (2015). The journey of DNA repair. Trends Cancer 1, 215–216. 10.1016/j.trecan.2015.11.001 26858989 PMC4743047

[B103] Salvi J. S. Mekhail K. (2015). R-loops highlight the nucleus in ALS. Nucleus 6, 23–29. 10.1080/19491034.2015.1004952 25587791 PMC4615755

[B104] Schellino R. Boido M. Vercelli A. (2019). JNK signaling pathway involvement in spinal cord neuron development and death. Cells 8:1576. 10.3390/cells8121576 31817379 PMC6953032

[B105] Schrank B. Gotz R. Gunnersen J. M. Ure J. M. Toyka K. V. Smith A. G.. (1997). Inactivation of the survival motor neuron gene, a candidate gene for human spinal muscular atrophy, leads to massive cell death in early mouse embryos. Proc. Natl. Acad. Sci. U S A 94, 9920–9925. 10.1073/pnas.94.18.9920 9275227 PMC23295

[B106] Sepe S. Payan-Gomez C. Milanese C. Hoeijmakers J. H. Mastroberardino P. G. (2013). Nucleotide excision repair in chronic neurodegenerative diseases. DNA Repair (Amst) 12, 568–577. 10.1016/j.dnarep.2013.04.009 23726220

[B107] Shababi M. Lorson C. L. Rudnik-Schoneborn S. S. (2014). Spinal muscular atrophy: a motor neuron disorder or a multi-organ disease? J. Anat. 224, 15–28. 10.1111/joa.12083 23876144 PMC3867883

[B108] Shao T. Pan Y. H. Xiong X. D. (2020). Circular RNA: an important player with multiple facets to regulate its parental gene expression. Mol. Ther. Nucleic Acids 23, 369–376. 10.1016/j.omtn.2020.11.008 33425494 PMC7779830

[B109] Shiloh Y. Rotman G. (1996). Ataxia-telangiectasia and the ATM gene: linking neurodegeneration, immunodeficiency and cancer to cell cycle checkpoints. J. Clin. Immunol. 16, 254–260. 10.1007/BF01541389 8886993

[B110] Simon C. M. Dai Y. Van Alstyne M. Koutsioumpa C. Pagiazitis J. G. Chalif J. I.. (2017). Converging mechanisms of p53 activation drive motor neuron degeneration in spinal muscular atrophy. Cell Rep. 21, 3767–3780. 10.1016/j.celrep.2017.12.003 29281826 PMC5747328

[B111] Singh R. N. Howell M. D. Ottesen E. W. Singh N. N. (2017). Diverse role of survival motor neuron protein. Biochim. Biophys. Acta 1860, 299–315. 10.1016/j.bbagrm.2016.12.008 28095296 PMC5325804

[B112] Skourti-Stathaki K. Proudfoot N. J. (2014). A double-edged sword: R loops as threats to genome integrity and powerful regulators of gene expression. Genes Dev. 28, 1384–1396. 10.1101/gad.242990.114 24990962 PMC4083084

[B113] Skourti-Stathaki K. Proudfoot N. J. Gromak N. (2011). Human senataxin resolves RNA/DNA hybrids formed at transcriptional pause sites to promote Xrn2-dependent termination. Mol. Cell 42, 794–805. 10.1016/j.molcel.2011.04.026 21700224 PMC3145960

[B114] Sollier J. Stork C. T. Garcia-Rubio M. L. Paulsen R. D. Aguilera A. Cimprich K. A.. (2014). Transcription-coupled nucleotide excision repair factors promote R-loop-induced genome instability. Mol. Cell 56, 777–785. 10.1016/j.molcel.2014.10.020 25435140 PMC4272638

[B115] Stathas D. Kalfakis N. Kararizou E. Manta P. (2008). Spinal muscular atrophy: DNA fragmentation and immaturity of muscle fibers. Acta Histochem. 110, 53–58. 10.1016/j.acthis.2007.06.001 17761239

[B116] Sun S. Ling S. C. Qiu J. Albuquerque C. P. Zhou Y. Tokunaga S.. (2015). ALS-causative mutations in FUS/TLS confer gain and loss of function by altered association with SMN and U1-snRNP. Nat. Commun. 6:6171. 10.1038/ncomms7171 25625564 PMC4338613

[B117] Suraweera A. Lim Y. Woods R. Birrell G. W. Nasim T. Becherel O. J.. (2009). Functional role for senataxin, defective in ataxia oculomotor apraxia type 2, in transcriptional regulation. Hum. Mol. Genet. 18, 3384–3396. 10.1093/hmg/ddp278 19515850

[B118] Takaku M. Tsujita T. Horikoshi N. Takizawa Y. Qing Y. Hirota K.. (2011). Purification of the human SMN-GEMIN2 complex and assessment of its stimulation of RAD51-mediated DNA recombination reactions. Biochemistry 50, 6797–6805. 10.1021/bi200828g 21732698

[B119] Takizawa Y. Qing Y. Takaku M. Ishida T. Morozumi Y. Tsujita T.. (2010). GEMIN2 promotes accumulation of RAD51 at double-strand breaks in homologous recombination. Nucleic Acids Res. 38, 5059–5074. 10.1093/nar/gkq271 20403813 PMC2926616

[B120] Tews D. S. Goebel H. H. (1996). DNA fragmentation and BCL-2 expression in infantile spinal muscular atrophy. Neuromuscul. Disord. 6, 265–273. 10.1016/0960-8966(96)00018-1 8887956

[B121] Ursic D. Chinchilla K. Finkel J. S. Culbertson M. R. (2004). Multiple protein/protein and protein/RNA interactions suggest roles for yeast DNA/RNA helicase Sen1p in transcription, transcription-coupled DNA repair and RNA processing. Nucleic Acids Res. 32, 2441–2452. 10.1093/nar/gkh561 15121901 PMC419450

[B122] Van Alstyne M. Simon C. M. Sardi S. P. Shihabuddin L. S. Mentis G. Z. Pellizzoni L.. (2018). Dysregulation of Mdm2 and Mdm4 alternative splicing underlies motor neuron death in spinal muscular atrophy. Genes Dev. 32, 1045–1059. 10.1101/gad.316059.118 30012555 PMC6075148

[B123] van der Lelij P. Oostra A. B. Rooimans M. A. Joenje H. De Winter J. P. (2010). Diagnostic overlap between fanconi anemia and the cohesinopathies: roberts syndrome and warsaw breakage syndrome. Anemia 2010:565268. 10.1155/2010/565268 21490908 PMC3065841

[B124] Veldink J. H. Kalmijn S. Van Der Hout A. H. Lemmink H. H. Groeneveld G. J. Lummen C.. (2005). SMN genotypes producing less SMN protein increase susceptibility to and severity of sporadic ALS. Neurology 65, 820–825. 10.1212/01.wnl.0000174472.03292.dd 16093455

[B125] Walker C. El-Khamisy S. F. (2018). Perturbed autophagy and DNA repair converge to promote neurodegeneration in amyotrophic lateral sclerosis and dementia. Brain 141, 1247–1262. 10.1093/brain/awy076 29584802 PMC5917746

[B126] Wang H. B. Dharmalingam P. Vasquez V. Mitra J. Boldogh I. Rao K. S.. (2017). Chronic oxidative damage together with genome repair deficiency in the neurons is a double whammy for neurodegeneration: is damage response signaling a potential therapeutic target? Mech. Ageing Dev. 161, 163–176. 10.1016/j.mad.2016.09.005 27663141 PMC5316312

[B127] Wang I. X. Grunseich C. Fox J. Burdick J. Zhu Z. Ravazian N.. (2018). Human proteins that interact with RNA/DNA hybrids. Genome Res. 28, 1405–1414. 10.1101/gr.237362.118 30108179 PMC6120628

[B128] Wang J. Haeusler A. R. Simko E. A. (2015). Emerging role of RNA*DNA hybrids in C9orf72-linked neurodegeneration. Cell Cycle 14, 526–532. 10.1080/15384101.2014.995490 25590632 PMC4614400

[B129] Wirth B. (2021). Spinal muscular atrophy: in the challenge lies a solution. Trends Neurosci. 44, 306–322. 10.1016/j.tins.2020.11.009 33423791

[B130] Wood M. Quinet A. Lin Y. L. Davis A. A. Pasero P. Ayala Y. M.. (2020). TDP-43 dysfunction results in R-loop accumulation and DNA replication defects. J. Cell Sci. 133:jcs244129. 10.1242/jcs.244129 32989039 PMC7648616

[B131] Wu C. Y. Whye D. Glazewski L. Choe L. Kerr D. Lee K. H.. (2011). Proteomic assessment of a cell model of spinal muscular atrophy. BMC Neurosci. 12:25. 10.1186/1471-2202-12-25 21385431 PMC3063191

[B132] Xu X. Zhang J. Tian Y. Gao Y. Dong X. Chen W.. (2020). CircRNA inhibits DNA damage repair by interacting with host gene. Mol. Cancer 19:128. 10.1186/s12943-020-01246-x 32838810 PMC7446195

[B133] Yuce O. West S. C. (2013). Senataxin, defective in the neurodegenerative disorder ataxia with oculomotor apraxia 2, lies at the interface of transcription and the DNA damage response. Mol. Cell Biol 33, 406–417. 10.1128/MCB.01195-12 23149945 PMC3554130

[B134] Zhang Z. Lotti F. Dittmar K. Younis I. Wan L. Kasim M.. (2008). SMN deficiency causes tissue-specific perturbations in the repertoire of snRNAs and widespread defects in splicing. Cell 133, 585–600. 10.1016/j.cell.2008.03.031 18485868 PMC2446403

[B135] Zhang Z. Pinto A. M. Wan L. Wang W. Berg M. G. Oliva I.. (2013). Dysregulation of synaptogenesis genes antecedes motor neuron pathology in spinal muscular atrophy. Proc. Natl. Acad. Sci. U S A 110, 19348–19353. 10.1073/pnas.1319280110 24191055 PMC3845193

[B136] Zhao D. Y. Gish G. Braunschweig U. Li Y. Ni Z. Schmitges F. W.. (2016). SMN and symmetric arginine dimethylation of RNA polymerase II C-terminal domain control termination. Nature 529, 48–53. 10.1038/nature16469 26700805

